# Supplementary material for: The Construction and Exploration of a Comprehensive MicroRNA Centered Regulatory Network in Foxtail Millet (Setaria italica L.)
Source: Front Plant Sci. 2022 May 6;13:848474. doi: 10.3389/fpls.2022.848474 (PMC9121102; doi:10.3389/fpls.2022.848474)
Supplement: Supplementary file 6 [file Data_Sheet_6.zip › Sit-miR528_Seita.4G077000.1_862_TPlot.pdf]

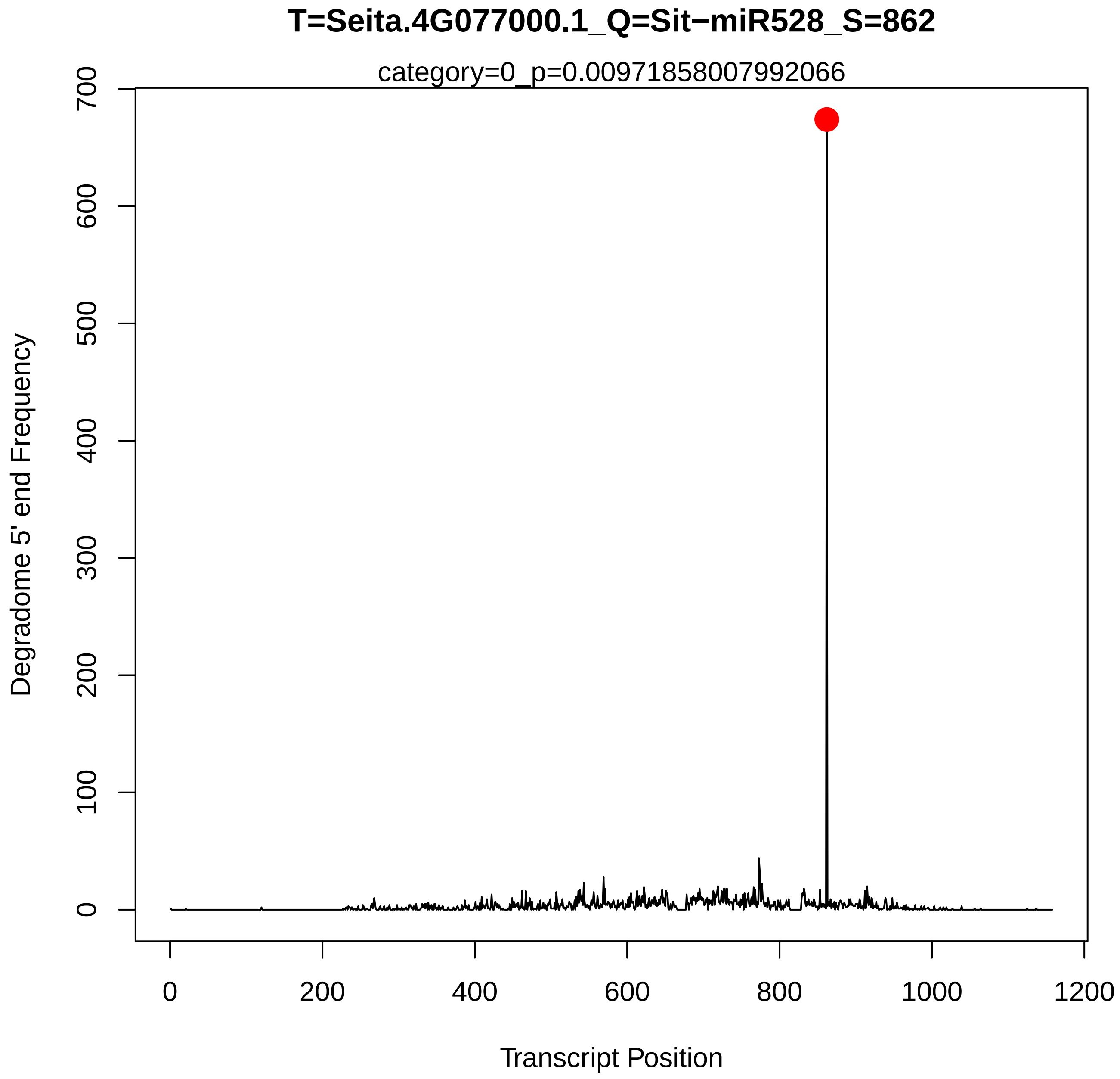
T=Seita.4G077000.1_Q=Sit−miR528_S=862
category=0_p=0.00971858007992066
Degradome 5' end Frequency
0
100
200
300
400
500
600
700
Transcript Position
0
200
400
600
800
1000
1200
